# Clinical effects of CYP2D6 phenoconversion in patients with psychosis

**DOI:** 10.1177/02698811241278844

**Published:** 2024-09-23

**Authors:** Emma Y De Brabander, Esmee Breddels, Therese van Amelsvoort, Roos van Westrhenen, GROUP Investigators

**Affiliations:** 1 Department of Psychiatry and Neuropsychology, Research Institute for Mental Health and Neuroscience, Maastricht University (Medical Center), Maastricht, The Netherlands; 2 Department of Cognitive Neuroscience, Donders Institute for Brain, Cognition, and Behaviour, Radboud University Medical Center, Nijmegen, The Netherlands; 3 Outpatient Clinic Pharmacogenetics, Parnassia Groep, Amsterdam, The Netherlands; 4 Institute of Psychiatry, Psychology, and Neurosciences, King’s College London, London, UK; 5 St. John’s National Academy of Health Sciences, Bangalore, India

**Keywords:** Pharmacogenetics, CYP2D6, drugs for psychosis management, psychosis, phenoconversion

## Abstract

**Background:**

Pharmacogenetics is considered a promising avenue for improving treatment outcomes, yet evidence arguing for the use of pharmacogenetics in the treatment of psychotic disorders is mixed and clinical usefulness is under debate. Many patients with psychosis use multiple medications, which can alter the metabolic capacity of *CYP* enzymes, a process called phenoconversion. In clinical studies, treatment outcomes of drugs for psychosis management may have been influenced by phenoconversion.

**Aim:**

Here we evaluate the impact and predictive value of CYP2D6 phenoconversion in patients with psychotic disorders under pharmacological treatment.

**Method:**

Phenoconversion-corrected phenotype was determined by accounting for inhibitor strength. Phenoconversion-corrected and genotype-predicted phenotypes were compared in association with side effects, subjective well-being and symptom severity.

**Results:**

Phenoconversion led to a large increase in poor metabolizers (PMs; 17–82, 16% of sample), due to concomitant use of the serotonin reuptake inhibitors fluoxetine and paroxetine. Neither CYP2D6-predicted nor phenoconversion-corrected phenotype was robustly associated with outcome measures. Risperidone, however, was most affected by the *CYP2D6* genotype.

**Conclusion:**

Polypharmacy and phenoconversion were prevalent and accounted for a significant increase in PMs. CYP2D6 may play a limited role in side effects, symptoms and well-being measures. However, due to the high frequency of occurrence, phenoconversion should be considered in future clinical trials.

## Background

Antipsychotics, or drugs used in psychosis management, are prescribed for a variety of disorders, most commonly psychotic disorders (Christian et al., 2012). Although pharmacological treatment was effective compared to placebo, treatment failure remains common (El Abdellati et al., 2020; Pandey and Kalita, 2022). Discontinuation or switching of medication may occur due to high frequency of or burdensome side effects, or because of a lack of perceived symptom improvement (Ascher-Svanum et al., 2010; Liu-Seifert et al., 2005; Read and Williams, 2019). It is estimated that pharmacological treatment fails in an estimated 30% of patients with schizophrenia starting psychotropic medication, demonstrating a clear need to improve patient care (Pandey and Kalita, 2022).

Heterogeneity at patient, disease and environmental levels is thought to contribute to a lack of response to medication. One source of heterogeneity arises from genes encoding the metabolic enzymes responsible for medication breakdown (van Westrhenen et al., 2020; Westrhenen et al., 2021). Common genetic variants can lead to increased or decreased enzyme activity, resulting in faster or slower metabolism of medication (Gaedigk et al., 2008). The hepatic cytochrome P450 (CYP) 2D6 is responsible for the metabolization of 20 drugs for psychosis (Drugbank) (https://go.drugbank.com/categories/DBCAT002623) and approximately 25% of all medications (Ingelman-Sundberg, 2005), making it a useful target for personalized medicine.

However, CYP2D6 is a complex and highly polymorphic target. To facilitate translation from genotype to phenotype and stimulate standardization, an activity score (AS) system has been proposed (Gaedigk et al., 2008, 2018). Here, each star-annotated variant encodes for a specific AS, ranging from 0 (no activity) to 1 (normal activity). Summing both allele-predicted AS leads to a predicted phenotype; poor metabolizer (PM) (AS = 0), intermediate metabolizer (IM) (AS = 0.5), normal metabolizer (NM) (AS = 1–2), or ultrarapid metabolizer (UM) (AS > 2, due to copy number variants) (Gaedigk et al., 2008, 2018; Molden and Jukić, 2021). Several groups, including the Dutch Pharmacogenetic Working Group (DPWG) and the Clinical Pharmacogenetics Implementation Consortium, have formulated pharmacogenetic dosing guidelines, including for drugs for psychosis and depression, and (among other pharmacogenes) *CYP2D6* (Beunk et al., 2023; Brouwer et al., 2022).

Although pharmacogenetics is suggested as potentially beneficial in clinical practice, its application is still limited. This may be partly explained by the theoretical nature and challenges that arise when working with individual patients with comorbidity and polypharmacy. Polypharmacy can result in phenoconversion; the change in *CYP* activity due to the use of exogenous substances such as alcohol, caffeine, tobacco or a concomitant medication that can affect *CYP* enzyme activity, resulting in a different phenotype than would be predicted based on the individual’s DNA. In drug–drug interaction studies, the substrate or ‘victim’ drug is one where the plasma concentrations are altered due to inhibition or induction of the ‘perpetrator’ drug, that is, the drug that affects the involved metabolic pathway (Tornio et al., 2019). The severity of phenoconversion depends on the strength and timing of the perpetrator’s drug (Shah and Smith, 2015). In psychiatry, simultaneous use of CYP2D6 substrates is not uncommon (Stassen et al., 2022b). The noradrenergic and dopaminergic drug bupropion, and the serotonin reuptake inhibitors (SERTs) fluoxetine and paroxetine are all strong inhibitors, effectively reducing any individual’s metabolic capacity. It is hypothesized that this may result in a situation where patients could be considered IM or PM when multiple medications metabolized through the same *CYP* are used, even when genetic testing indicates they are NM individuals (Cicali et al., 2021). As a result, the concentration of the *CYP2D6*-metabolized medication exceeds what would be expected based on genotype, increasing the risk of adverse drug effects (Shah and Smith, 2015). Since the prescription of drugs for depression in combination with drugs for psychosis is increasingly common (Mao and Zhang, 2015), it would be advantageous to consider phenoconversion in pharmacogenetic studies and it should always be part of clinical application.

Not all psychosis treatment drugs would be affected by phenoconversion of CYP2D6, as there are differences in metabolic pathways and involvement of CYP2D6. To be affected by *CYP2D6* and possible phenoconversion, it should be a major substrate of the enzyme. This is the case for example, haloperidol, risperidone and aripiprazole, but not for example, quetiapine and olanzapine (Samer et al., 2013). The DPWG guidelines only recommend dose alterations due to CYP2D6 for aripiprazole, brexiprazole, haloperidol, pimozide, risperidone and zuclopenthixol (Beunk et al., 2023). Furthermore, current evidence on the effect of non-NM phenotype in users of drugs for psychosis is mixed (Austin-Zimmerman et al., 2021; Calafato et al., 2020; Dorado et al., 2007; Fleeman et al., 2011; Maruf et al., 2021; Wannasuphoprasit et al., 2021; Zhang and Malhotra, 2011). Proper examination of phenoconversion is often lacking, which may contribute to conflicting results as patients can be misclassified into a metabolizer group based on only their genotype. Thus, there is a need for representation of the real-world value of pharmacogenetics including phenoconversion.

Here, we retrospectively evaluated the frequency and impact of phenoconversion in a relatively large Dutch cohort of patients with psychosis (GROUP cohort; Korver et al. (2012)). As phenoconversion-corrected phenotype (pPT) could be a more accurate representation of the actual metabolic capacity affecting outcome compared to genotype-predicted phenotype (gPT), we hypothesize it may also be more closely associated with symptom severity, side effects and well-being of patients measured using questionnaires. The effect of CYP2D6 is expected only for substrate (‘victim’) drugs, whereas drugs metabolized primarily through other routes should remain largely unaffected.

As an exploratory analysis, we also examined phenoconversion in the sample stratified by sex. Sex is often included as a covariate, correcting for any differences that may be related to sex. As a result, information on sex-related differences may be lost. With this, we aim to contribute to a better understanding of differences between men and women in the influence and impact of *CYP2D6* phenotype and phenoconversion.

## Methods

### Study design and participants

A retrospective design was employed using the baseline data from the prospective Genetic Risk And Outcome of Psychosis (GROUP) cohort, the details of which have been described elsewhere (Korver et al., 2012). Patients were identified through clinicians active in local psychosis departments or academic centres in the Netherlands and (Dutch speaking part of) Belgium. Both in- and out-patients were eligible. Inclusion criteria were as follows: (1) between 16 and 50 years of age; (2) diagnosed with non-affective psychotic disorder according to the Diagnostic and Statistical Manual of Mental Disorders, Fourth Edition (DSM-IV) (American Psychiatric Association, 2000), with a maximum period of time since first contact with mental health care of 5 years; (3) good command of the Dutch language; and (4) able and willing to give written informed consent. The study was conducted in accordance with the Declaration of Helsinki (World Medical Association, 2004, 2013) and the study protocol was approved by the Ethical Review Board of the University Medical Centre Utrecht centrally (protocol code 04/003, 13 May 2004), and by each local review board per participating institute. Patients who were taking at least one drug for psychosis as a treatment drug at baseline, and for whom gPT was available, were included in the current study. Patients who had an unknown phenotype or who reported chlorpromazine-equivalent dose of their main treatment drug of below 25 mg or above 1000 mg per day were excluded.

### Measurements

#### Outcome

For each outcome, data collected at baseline were used.

##### Abnormal Involuntary Movement Scale (AIMS)

Abnormal movements in the facial and oral area, trunk, and extremities are measured by the clinician or research team, to form an assessment of the extrapyramidal symptom of dyskinesia, together with a global assessment (Guy, 1976).

##### Barnes Akathisia Rating Scale (BARS)

Akathisia presents as motor restlessness, part of the extrapyramidal symptom group. The BARS is a commonly used scale to measure clinical features of akathisia based on clinician observation, patient report of restlessness, and patient report of distress due to restlessness, amounting to a total score to indicate an overall assessment of akathisia (Barnes, 2003).

##### The Unified Parkinson’s Disease Rating Scale (UPDRS)

Parkinsonian movements can be characterized by slowed movements, rigidity and tremors. The UPDRS is a valid and widely used instrument in Parkinson’s disease research and provides another measure of extrapyramidal symptoms (Martínez-Martín et al., 1994).

##### The Subjective Well-being Under Neuroleptics Scale (SWN20)

The SWN20 provides a measure of subjective well-being using five subscales: emotional regulation, mental functioning, physical functioning, social integration and self-control (Naber et al., 1994, 2001).

##### Positive and Negative Syndrome Scale (PANSS)

The PANSS combines the Brief Psychiatric Rating Scale and Psychopathology Rating Schedule to assess positive and negative symptoms of schizophrenia as well as general psychopathology using a 30-item questionnaire. Scale-specific items are summed to calculate a score on the positive and negative symptom subscales, which have been shown to have strong reliability, validity and sensitivity (Kay et al., 1987; Leucht et al., 2005; Peralta and Cuesta, 1994).

#### Covariates

Sex, age, ethnicity, number of psychotic episodes, illness duration in years, chlorpromazine-equivalent dose of the main treatment drug, total number of treatment (for psychosis) drugs prescribed, total number of medications prescribed, smoking status and hormonal birth control status were included as covariates. Body mass index (BMI) was not available or computable for this time point and was therefore not included as a covariate.

#### Genotyping

From each subject, 20 ml of blood was collected at the participating mental health institutes. The blood sample was sent to the University Medical Centre Utrecht by mail, where DNA extraction was performed from peripheral blood lymphocytes. Quality control procedures were performed using PLINK v1.9 according to standard protocol (Purcell and Chang; Purcell et al., 2007). Genotyping and quality control details have been described elsewhere (Sandhu et al., 2023) and in the Supplemental material. For an extensive overview of genotype–phenotype translation of *CYP2D6*, we also refer to the supplemental material of Sandhu et al. (2023). Briefly, genotypes of 570,038 single-nucleotide variants were characterized for 2818 individuals (including patients, siblings, parents, and healthy controls), using a customized Illumina Institute of Psychological Medicine and Clinical Neurology chip array and were subjected to an extensive quality control procedure. Non-genotyped variants were imputed using the 1000 Genome Phase 3 (v5) reference panel using the Michigan Imputation Server (Das et al., 2016). A post-imputation filter of R2 > 0.5 was applied to include only high-quality rare variants. Genotype-predicted UM could not be assessed as structure variants (including copy number variants) were not included in the GWAS panels.

#### *CYP2D6* phenotype prediction and phenoconversion calculation

Stargazer, a python-based bioinformatics tool, was used to predict metabolizer groups of *CYP2D6* (Lee et al., 2019a, 2019b). Imputed data for chromosome 22 were used to identify *CYP2D6* star alleles (haplotypes), and an AS was generated accordingly. A functional phenotype classification per individual was generated. To improve accuracy, phenotype classifications were cross-checked with the PharmVar genotype–phenotype translation tables and the Pharmacogenomics Knowledgebase (PharmVar, 2022; PharmGKB), as explained in more detail by Sandhu et al. (2023).

Concurrent *CYP2D6*-inhibitor use was checked per patient. First, all recorded drug information was checked and standardized (i.e. medication names were converted to identical spelling of the generic name, and entries with missing or unclear information on medication type, use, or dose were removed). Known *CYP2D6* inhibitors were marked according to strength: weak, moderate and strong, according to Flockhart et al. (2021). Inducers were not included, since there are no known *CYP2D6* inducers (see Supplemental Table S1). Following this, a new variable representing pPT was created. All individuals were assigned a pPT. pPT was the same as gPT if the patient was not using any *CYP2D6* inhibitors (e.g. the pPT would be NM if gPT was NM and the patient did not use any inhibitors) and was adjusted to a reduced metabolism type according to the methods of Cicali et al. (2021), accounting for inhibitor strength. Weak inhibitors do not cause phenoconversion, while strong inhibitors such as paroxetine and fluoxetine have previously been shown to reduce most individuals to PM (Bahar et al., 2018; Storelli et al., 2018). Although true conversion to PM can only be confirmed using serum measurements, we used the method of Cicali et al. (2021), that is, assigning a pPT based on inhibitor strength, to represent clinical practicality as most clinicians will not have serum measurements available. Thus, individuals taking a strong inhibitor were assigned a poor pPT (pPM) in all cases (i.e. NM and IM individuals were assigned pPM. PM individuals cannot be further reduced, and would remain pPM). If an individual was only taking a weak inhibitor, the pPT remained the same as their gPT. Moderate inhibitors would only affect normal gPT (gNM) individuals, reducing their status to intermediate (pIM), but not reduce intermediate metabolizers (gIM) to pPM. If individuals were taking multiple inhibitors, the highest strength was used to assign pPT. Figure S1 (Supplemental material) provides an overview.

#### Statistical analysis

Between-group differences between phenotype groups and between the most commonly reported drug subsamples were examined using Kruskal-Wallis and Dunn tests (for continuous variables) and Fisher’s exact and/or Pearson’s Chi-square tests (for categorical variables). Both differences between gPT groups and pPT groups were examined. Statistical analyses were performed using R version 4.2.3 (R Core Team, 2023).

To investigate whether pPT is more strongly associated with each outcome compared to gPT, regression models were generated and compared to find the best model, for the most used drugs separately. Linear models were used for continuous outcomes (SWN20, AIMS, UPDRS, PANSS), and a proportional odds cumulative logit model for categorical (BARS), as the BARS has six outcomes (none, minimal, mild, moderate, severe and extreme). Multiple models were explored per outcome, using a backward stepwise variable selection method. This was done twice, once using gPT as a predictor, and once with pPT. The best-fit model per outcome was selected using the Akaike information criterion (AIC) (Bozdogan, 1987) and *R*^2^ (Wright, 1921). The AIC is an information criteria-based relative fit index developed as an estimation of the accuracy of a model to predict outcomes in samples other than the provided data, and *R*^2^ represents the percentage of total variation in the outcome that is explained by the predictors. According to the AIC rule of thumb, if the difference in AIC between two models is less than two, this suggests the models are indistinguishable.

The ‘stats’ package was used for the Mann–Whitney *U* test, Chi-square test, Kruskal–Wallis rank sum test, Fisher’s exact test for count data, and general linear models (R Core Team, 2023). Dunn tests were performed using the *dunn_test()* function from the ‘rstatix’ package (Kassambra, 2023). The ‘VGAM: Vector Generalized Linear and Additive Models’ package provided the *vglm()* function for multinomial models. The *step4vglm()* function from this package was used to select the final best multinomial model, using backward direction and specifying the genotype variables to remain included using the scope argument of this function (Yee, 2010). Similarly, stepAIC() from the ‘MASS’ package was used to select the best-fitting GLM models (Venables and Ripley, 2002). AIC was automatically compared across models per outcome using the *aictab()* function from the ‘AICcmodavg’ package (Mazerolle, 2023).

A *p-*value of <0.05 was considered statistically significant. All reported *p*-values are corrected for multiple testing using the Bonferroni method. GROUP release number 8.0 was used for the current analysis.

## Results

### Patient characteristics

In all, 412 patients were included (79.6% male, mean age 27 ± 7.1 years). In total, 228 patients (55.3%) were genotype-predicted NMs (gNM), 167 (40.5%) were IMs (gIM) and 17 (4.1%) were PMs (gPM). Six patients (not included in the described sample) were classified as ‘unknown’ metabolizers and were not included in the analysis. A Chi-square test confirmed this distribution was not different than would be expected in a European population based on existing literature (Gaedigk et al., 2017) (*p* = 0.21). See Table S1 for an overview of patient characteristics of the complete sample.

Risperidone (*n* = 111), olanzapine (*n* = 122), clozapine (*n* = 60) and aripiprazole (*n* = 49) were the four most reported treatment drugs and were thus selected to investigate the association between genotype prediction and outcome. Tables 1–5 provide demographic information on this sample and per drug group. Risperidone and aripiprazole are both metabolized through *CYP2D6* to active metabolites 9-hydroxyrisperidone (risperidone) and dehydroaripiprazole (aripiprazole) and *CYP2D6* inhibition has been shown to increase relative plasma concentration (Fang et al., 1999; Molden et al., 2006). By contrast, neither olanzapine nor clozapine is extensively metabolized through *CYP2D6* (Samer et al., 2013). Due to the differential effects of *CYP2D6* inhibitors on each drug, each group was analysed separately. For example, *CYP2D6* activity is strongly correlated with risperidone/9-hydroxyrisperidone ratio, but this relationship is less clear for aripiprazole/dehydroaripiprazole and the expected effect on efficacy is uncertain (Berecz et al., 2004; Zhang et al., 2019).

**Table 1.** Demographic variables. Between-drug differences are marked with two asterisks at *p* < 0.01 and with a single asterisk at *p* < 0.05.

Demographic variables	Total	Men	Women
Patients (% of total)	342 (100%)	282 (82.5%)	60 (17.5%)
Average age in years**	27.1 (±6.9)	26.6 (±6.09)	29.5 (±9.9)
Average number of total medications**	3.24 (±2.04)	3.2 (±2.02)	3.6 (±2.2)
Average duration of illness in years**	4.3 (±3.5)	4.36 (±3.5)	4 (±3.4)
Number of psychotic episodes	1.7 (±1.03)	1.7 (±1.03)	1.8 (±1)
Ethnicity			
White	281 (82.2%)	231 (81.9%)	50 (83.3%)
Moroccan	6 (1.8%)	6 (2.1%)	0
Turkish	10 (2.9%)	10 (3.5%)	0
Other	8 (2.3%)	7 (2.5%)	1 (1.7%)
Mixed	33 (9.6%)	25 (8.9%)	8 (13.3%)
Not reported	4 (1.2%)	3 (1.1%)	1 (1.7%)
Diagnosis			
Schizophrenia and related disorders (including schizophreniform and schizoaffective)	295 (86.3%)	246 (87.2%)	49 (81.7%)
Bipolar disorder	1 (0.3%)	1 (0.4%)	0
Delusional disorder	9 (2.6%)	7 (2.4%)	2 (3.3%)
Psychosis	36 (10.5%)	27 (9.6%)	9 (15%)
Unknown	1 (0.3%)	1 (0.4%)	0
Smoking status			
Smoker	121 (35.4%)	87 (30.9%)	34 (56.7%)
Non-smoker	215 (62.9%)	190 (67.4%)	25 (41.7%)
Unknown	6 (1.7%)	5 (1.7 %)	1 (1.6%)
Reported use of birth control (oral birth control and IUD)**	22 (6.4%)	NA	22 (36.7%)
Main treatment drug			
Olanzapine	122 (35.7%)	101 (35.8%)	21 (35%)
Risperidone	111 (32.5%)	91 (32.3%)	20 (33.3%)
Clozapine	60 (17.5%)	53 (18.8%)	7 (11.7%)
Aripiprazole	49 (14.3%)	37 (13.1%)	12 (20%)
Chlorpromazine-equivalent dose of main treatment drug (in mg)**	367.6 (±193.3)	382 (±195)	300 (±171)
*CYP2D6* inhibitors (strength)			
Paroxetine (strong)	49 (14.3%)	38 (13.5%)	11 (18.3%)
Citalopram (weak)	20 (5.9%)	18 (6.4%)	2 (3.3%)
Fluoxetine (strong)	10 (2.9%)	8 (2.8%)	2 (3.3%)
Sertraline (weak)	10 (2.9%)	8 (2.8%)	2 (3.3%)
Escitalopram (weak)	5 (1.5%)	4 (1.2%)	1 (1.7%)
Clomipramine (weak)	2 (0.6%)	1 (0.4%)	1 (1.7%)
Levomepromazine (weak)	1 (0.3%)	0	1 (1.7%)
*CYP2D6* genotype-predicted phenotype			
PM	16 (4.7%)	12 (4.2%)	4 (6.7%)
IM	130 (38%)	104 (36.9%)	26 (43.3%)
NM	196 (57.3%)	166 (58.9%)	30 (50%)
*CYP2D6* phenoconversion-corrected phenotype			
PM	67 (19.6%)	52 (18.4%)	15 (25%)
IM	101 (29.5%)	80 (28.4%)	21 (35%)
NM	174 (50.9%)	150 (53.2%)	24 (40%)

PM: poor metabolizer; IM: intermediate metabolizer; NM: normal metabolizer.

**Table 2.** Demographic variables for the risperidone group.

Demographic variables	Total	Men	Women
Patients (% of total)	111 (100%)	91 (82%)	20 (18%)
Average age in years	24.9 (±6.5)	24.9 (±5.7)	25.2 (±9.8)
Average number of total medications	2.9 (±1.9)	2.7 (±1.7)	3.8 (±2.5)
Average duration of illness in years	3.1 (±2.9)	3.3 (±3.1)	2.5 (±2.2)
Number of psychotic episodes	1.6 (±1.1)	1.7 (±1.2)	1.4 (±0.7)
Ethnicity			
White	96 (86.5%)	77 (84.6%)	19 (95%)
Moroccan	1 (0.9%)	1 (1.1%)	0
Turkish	2 (1.8%)	2 (2.2%)	0
Other	5 (4.5%)	4 (4.4%)	1 (5%)
Mixed	7 (6.3%)	7 (7.7%)	0
Diagnosis			
Schizophrenia and related disorders (including schizophreniform and schizoaffective)	86 (77.5%)	74 (81.3%)	12 (60%)
Delusional disorder	6 (5.4%)	4 (4.4%)	2 (10%)
Psychosis	19 (17.1%)	13 (14.3%)	6 (30%)
Smoking status			
Smoker	77 (69.4%)	67 (73.6%)	10 (50%)
Non-smoker	33 (29.7%)	23 (25.3%)	10 (50%)
Unknown	1 (0.9%)	1 (1.1%)	0
Reported use of birth control (oral birth control and IUD)	6 (5.4%)	NA	6 (30%)
Chlorpromazine-equivalent dose (in mg)	288 (±159)	296 (±160)	248 (±150)
*CYP2D6* inhibitors (strength)			
Paroxetine (strong)	12 (10.9%)	7 (7.7%)	5 (25%)
Citalopram (weak)	5 (4.5%)	4 (4.4%)	1 (5%)
Fluoxetine (strong)	3 (2.7%)	1 (1.1%)	2 (10%)
Sertraline (weak)	5 (4.5%)	3 (3.3%)	2 (10%)
Escitalopram (weak)	1 (0.9%)	1 (1.1%)	0
*CYP2D6* genotype-predicted phenotype			
PM	5 (4.5%)	3 (3.3%)	2 (10%)
IM	42 (37.9%)	31 (34.1%)	11 (55%)
NM	64 (57.6%)	57 (62.6%)	7 (35%)
*CYP2D6* phenoconversion-corrected phenotype			
PM	17 (15.3%)	10 (11%)	7 (35%)
IM	38 (34.2%)	27 (29.7%)	11 (55%)
NM	56 (50.5%)	54 (59.3%)	2 (10%)

PM: poor metabolizer; IM: intermediate metabolizer; NM: normal metabolizer.

**Table 3.** Demographic variables for the olanzapine group.

Demographic variables	Total	Men	Women
Patients (% of total)	122 (100%)	101 (82.8%)	21 (17.2%)
Average age in years	28.7 (±7.8)	27.6 (±6.3)	34.3 (±10.9)
Average number of total medications	2.9 (±2)	2.9 (±2)	2.9 (±1.8)
Average duration of illness in years	4.3 (±4)	4.3 (±3.8)	4.2 (±4.7)
Number of psychotic episodes	1.6 (±0.9)	1.6 (±0.9)	1.6 (±1.1)
Ethnicity			
White	100 (83%)	84 (83.2%)	16 (76.2%)
Moroccan	4 (3.2%)	4 (4%)	0
Turkish	4 (3.2%)	4 (4%)	0
Other	2 (1.6%)	2 (2%)	0
Mixed	11 (9%)	7 (6.8%)	5 (23.8%)
Diagnosis			
Schizophrenia and related disorders (including schizophreniform and schizoaffective)	108 (88.5%)	88 (87.1%)	20 (95.2%)
Bipolar disorder	1 (0.8%)	1 (1%)	0
Delusional disorder	2 (1.6%)	2 (2%)	0
Psychosis	10 (8.3%)	9 (8.9%)	1 (4.8%)
Unknown	1 (0.8%)	1 (1%)	0
Smoking status			
Smoker	77 (63.1%)	68 (67.3%)	9 (42.9%)
Non-smoker	43 (35.3%)	31 (30.7%)	12 (57.1%)
Unknown	2 (1.6%)	2 (2%)	0
Reported use of birth control (oral birth control and IUD)	7 (5.7%)	NA	7 (33.3%)
Chlorpromazine-equivalent dose (in mg)	396 (±193)	409 (±189)	336 (±205)
*CYP2D6* inhibitors (strength)			
Paroxetine (strong)	16 (13.1%)	13 (12.9%)	3 (14.3%)
Citalopram (weak)	3 (2.5%)	3 (2.9%)	0
Fluoxetine (strong)	5 (4.1%)	5 (4.9%)	0
Sertraline (weak)	4 (3.3%)	4 (4%)	0
Escitalopram (weak)	1 (0.8%)	1 (1%)	0
Clomipramine (weak)	1 (0.8%)	1 (1%)	0
*CYP2D6* genotype-predicted phenotype			
PM	7 (5.7%)	7 (6.9%)	0
IM	38 (31.2%)	30 (29.7%)	8 (38%)
NM	77 (63.1%)	64 (63.4%)	13 (62%)
*CYP2D6* phenoconversion-corrected phenotype			
PM	25 (20.5%)	22 (21.8%)	3 (14.3%)
IM	27 (22.1%)	22 (21.8%)	5 (23.8%)
NM	70 (57.4%)	57 (56.4%)	13 (61.9%)

PM: poor metabolizer; IM: intermediate metabolizer; NM: normal metabolizer.

**Table 4.** Demographic variables for the clozapine group.

Demographic variables	Total	Men	Women
Patients (% of total)	60 (100%)	53 (88.3%)	7 (11.7%)
Average age in years	27.4 (±5.4)	27.9 (±5.5)	24 (±3.1)
Average number of total medications	4.3 (±2.2)	4.4 (±2.2)	4 (±2)
Average duration of illness in years	6.2 (±2.8)	6.1 (±2.9)	6.1 (±2.2)
Number of psychotic episodes	1.9 (±1.1)	1.8 (±1.1)	2.6 (±1.1)
Ethnicity			
White	46 (76.7%)	41 (77.4%)	5 (71.4%)
Moroccan	1 (1.7%)	1 (1.9%)	0
Turkish	3 (5%)	3 (5.6%)	0
Other	3 (5%)	2 (3.8%)	1 (14.3%)
Mixed	7 (11.6%)	6 (11.3%)	1 (14.3%)
Diagnosis			
Schizophrenia and related disorders (including schizophreniform and schizoaffective)	57 (95%)	51 (96.2%)	6 (85.7%)
Bipolar disorder	1 (1.67%)	0	1 (14.3%)
Delusional disorder	1 (1.67%)	1 (1.9%)	0
Psychosis	1 (1.67%)	1 (1.9%)	0
Smoking status			
Smoker	35 (58.3%)	33 (37.7%)	2 (28.6%)
Non-smoker	25 (41.7%)	20 (62.3%)	5 (71.4%)
Unknown	0	0	0
Reported use of birth control (oral birth control and IUD)	0 (0%)	NA	0 (0%)
Chlorpromazine-equivalent dose (in mg)	466 (±211)	478 (±209)	375 (±221)
*CYP2D6* inhibitors (strength)			
Paroxetine (strong)	10 (16.7%)	10 (18.9%)	0
Citalopram (weak)	4 (6.7%)	4 (7.5%)	0
Fluoxetine (strong)	1 (1.7%)	1 (1.9%)	0
Sertraline (weak)	1 (1.7%)	1 (1.9%)	0
Escitalopram (weak)	1 (1.7%)	1 (1.9%)	0
*CYP2D6* genotype-predicted phenotype			
PM	1 (1.7%)	0	1 (14.3%)
IM	26 (43.3%)	25 (47.2%)	1 (14.3%)
NM	33 (55%)	28 (52.8%)	5 (71.4%)
*CYP2D6* phenoconversion-corrected phenotype			
PM	11 (18.3%)	10 (18.9%)	1 (14.3%)
IM	19 (31.7%)	18 (34%)	1 (14.3%)
NM	30 (50%)	25 (47.1%)	5 (71.4%)

PM: poor metabolizer; IM: intermediate metabolizer; NM: normal metabolizer.

**Table 5.** Demographic variables for the aripiprazole group.

Demographic variables	Total	Men	Women
Patients (% of total)	49 (100%)	37 (75.5%)	12 (24.5%)
Average age in years	27.9 (±6.5)	26.7 (±6.2)	31.6 (±6.2)
Average number of total medications	3.7 (±1.9)	3.4 (±1.8)	4.4 (±2)
Average duration of illness in years	4.8 (±2.9)	4.8 (±3.2)	4.9 (±2)
Number of psychotic episodes	1.8 (±1.1)	1.7 (±1.1)	2.2 (±0.9)
Ethnicity			
White	39 (79.6%)	29 (78.4%)	10 (83.3%)
Turkish	1 (4.1%)	1 (2.7%)	0
Other	2 (2%)	2 (5.4%)	0
Mixed	7 (14.3%)	5 (13.5%)	2 (16.7%)
Diagnosis			
Schizophrenia and related disorders (including schizophreniform and schizoaffective)	43 (87.8%)	33 (89.2%)	10 (83.3%)
Psychosis	6 (12.2%)	4 (10.8%)	2 (16.7%)
Smoking status			
Smoker	26 (53.1)	22 (59.5%)	4 (33.4%)
Non-smoker	20 (40.8%)	13 (35.1%)	7 (58.3%)
Unknown	3 (6.1%)	2 (5.4%)	1 (8.3%)
Reported use of birth control (oral birth control and IUD)	8 (16.3%)	NA	8 (66.7%)
Chlorpromazine-equivalent dose (in mg)	357 (±173)	383 (±191)	278 (±52.2)
*CYP2D6* inhibitors (strength)			
Paroxetine (strong)	11 (22.5%)	8 (21.6%)	3 (25%)
Citalopram (weak)	8 (16.3%)	7 (18.9%)	1 (8.3%)
Fluoxetine (strong)	1 (2%)	1 (2.7%)	0
Escitalopram (weak)	2 (4.1%)	1 (2.7%)	1 (8.3%)
Clomipramine (weak)	1 (2%)	0	1 (8.3%)
Levomepromazine (weak)	1 (2%)	0	1 (8.3%)
*CYP2D6* genotype-predicted phenotype			
PM	3 (6.2%)	2 (5.4%)	1 (8.3%)
IM	24 (48.9%)	18 (48.6%)	6 (50%)
NM	22 (44.9%)	17 (46%)	5 (41.7%)
*CYP2D6* phenoconversion-corrected phenotype			
PM	14 (28.6%)	10 (27%)	4 (33.3%)
IM	17 (34.7%)	13 (35.1%)	4 (33.3%)
NM	18 (36.7%)	14 (37.9%)	4 (33.3%)

PM: poor metabolizer; IM: intermediate metabolizer; NM: normal metabolizer.

### Frequency of *CYP2D6* inhibitors and phenoconversion (complete sample)

In all, 102 patients were simultaneously using one or more *CYP2D6* inhibitor(s), resulting in 65 instances of phenoconversion (73.8% male, mean age 28.4 ± 7.8). There were 201 (48.8%) phenoconverted NMs (pNM), 129 (31.3%) phenoconverted IMs (pIM) and 82 (19.9%) phenoconverted PMs (pPM). Concomitantly used *CYP2D6* inhibitors were paroxetine (60 instances, 47.2%), citalopram (28 instances, 22%), fluoxetine (14 instances, 11%), sertraline (13 instances, 10.2%), escitalopram (7 instances, 5.5%), clomipramine (4 instances, 3.1%) and levomepromazine (one instance, 0.8%). Overall, 24 patients used two or more inhibitors, and the remaining 78 used one. Phenoconversion occurred mainly due to strong inhibitors paroxetine and fluoxetine. In all, 38 gIM and 27 gNM were converted to pPM due to the use of strong *CYP2D6* inhibitors. This is a 382% increase in PM (*n* = 17 prior to phenoconversion, *n* = 82 following phenoconversion) and 15.8% of the total sample.

When phenoconversion for *CYP2D6* was compared between men and women, 6% of women compared to 3.7% of men had gPM. The gIM phenotype was more prevalent in women compared to men (48.8% vs 38.4%*)*, but not significant (*p* = 0.16). In total, 29.8% of women were concomitantly using a *CYP2D6* inhibitor, versus 23.5% of men (*p* = 0.36). Although the distribution of inhibitor strength was the same for both sexes (~64% to 69% strong and ~31% to 36% weak), phenoconversion occurred in 20.2% of women compared to 14.6% of men (*p* = 0.28). When considering phenoconversion, 26.2% of women was pPM, 36.9% pIM and 36.9% pNM. In comparison, 18.3% of men were pPM, 38.4% pIM and 51.8% pNM. Women were at a 1.4 times higher risk for phenoconversion compared to men. Although the mean total medications was slightly lower in women versus men (3.2 and 3.7, respectively), the most commonly measured number of total medications for women was four, compared to two for men. Women were also at 1.3 times higher risk of taking concomitant medication in general, such as serotonin inhibitors, benzodiazepines and other drugs for psychosis. It should be noted, however, that there were no significant differences between sexes in medication use or phenoconversion rate (Table S4).

### Subgroups and between-group comparison

In the smaller group containing only the four most common drugs for psychosis, 80 patients reported using one or more *CYP2D6* inhibitor(s), leading to 51 cases of phenoconversion (78.4% male, mean age 27.8 ± 7.4). The percentage of patients per drug group who were phenoconverted to pPM was not significantly different between groups (*p* = 0.277) and ranged from 10.8% (12 individuals; risperidone group) to 22.4% (11 individuals; aripiprazole group). Phenoconversion to pPM occurred at a similar rate in the olanzapine and clozapine groups (14.7%/18 individuals in the olanzapine group; 16.7%/10 individuals in the clozapine group).

Age, chlorpromazine-equivalent dose, illness duration, number of treatment drugs for psychosis and medications total, and use of birth control differed between drug groups (see Table S2). Users of risperidone were younger and were prescribed lower chlorpromazine-equivalent doses. Similarly, the aripiprazole dose was lower compared to clozapine. Clozapine and aripiprazole users had a longer illness duration, although the number of psychotic episodes did not differ between groups. Risperidone users reported a higher concomitant use of other drugs for psychosis, but not concomitant medication in general. Users of either clozapine or aripiprazole reported taking more medications compared to risperidone and olanzapine users. Last, birth control use differed between aripiprazole and clozapine users. However, there were no birth control users in the clozapine group, and even though the reported use of birth control was proportionally highest in the aripiprazole group (16%), there were still only eight individuals.

When both sexes and all drugs were aggregated into one sample of *n* = 342, the PANSS positive scale was significantly different between pPT groups (*p* = 0.0285), where the pIM group had lower PANSS positive scores compared to the pPM group. When stratified by drug, the same result was found in the risperidone group only (*p* = 0.0286). Separated by sex, SWN20 scores were significantly lower for male pPM individuals compared to pNM over all drugs (*p* = 0.0461), but no drug-specific differences were found. For women, UPDRS scores were slightly higher for NM women using olanzapine in both phenotype groups (p/gNM compared to pPM; *p* = 0.0476 and compared to gIM; *p* = 0.0142. It should be noted that there were no gPM women using olanzapine). In the same group, gIM women had higher BARS scores compared to gNM (*p* = 0.0307).

### Association between genotype prediction and outcome

Predictors were first checked for multicollinearity, but no variables reached variance inflation factor >2. For each model, only complete cases per drug group were used (see Table S5). The model analysis was not repeated segregated by sex due to the small sample size per drug for women.

The final models for each outcome in each drug group are presented in Tables 6 and 7, separated by the role of *CYP2D6* in metabolism. AIC and *R*^2^ scores are shown in Table S6. With the exception of the risperidone group, pPT was included in most outcome models instead of gPT. The AIMS outcome model included pPT in all groups, and the UDPRS included gPT in all groups. It should be noted that the gPT and pPT models for most outcomes demonstrated similar AIC (difference of <2 points). This was not the case in the risperidone group for PANSS positive and UDPRS scores (AIC difference of 4.79 and 5.19, respectively); in the olanzapine group for PANSS positive and BARS scores (difference of 2.24 and 7.48, respectively); in the clozapine group for AIMS and SWN20 scores (difference of 3.36 and 2.87, respectively); and in the aripiprazole group for PANSS negative and UPDRS scores (difference of 2.1 and 2.17, respectively). In the risperidone, olanzapine and clozapine groups, phenotype was significantly associated with outcome. For risperidone, gPM individuals had significantly higher PANSS negative (*p* *=* 0.014) and UPDRS (*p* = 0.015) scores, but lower PANSS positive scores compared to gNM individuals (*p* = 0.015). pNM clozapine users had lower AIMS scores compared to pIM (*p* = 0.03), whereas gNM olanzapine users had better BARS outcomes compared to gIM (*p* = 0.003).

**Table 6.** Predictors included in final models for *CYP2D6* victim drugs risperidone and aripiprazole. Predictors with *p* < 0.01 are in italics and are marked with two asterisks, predictors with *p* < 0.05 have a single asterisk. Predictors with *p* < 0.1 are in italics only. Predictors with *p* > 0.1 are in standard lettering, without effect coefficients.

	Risperidone	Effect size (95% CI)	*p*-Value	Aripiprazole	Effect size (95% CI)	*p*-Value
AIMS	pPT, illness duration in years**, number of psychotic episodes, smoking status	0.03 (0.01; 0.049), −0.12 (−0.24; 4e-05)	0.003, 0.053	pPT, illness duration in years*, number of psychotic episodes*, ethnicity*, use of birth control*	−0.011 (−0.02; −0.001), 0.032 (0.006; 0.057), 0.01 (0.001; 0.019), 0.077 (0.01; 0.14)	0.03, 0.021, 0.037, 0.031
BARS	gPT. Chlorpromazine equivalent dose	−0.002 (−0.005; 4e-04)	0.092	gPM. Chlorpromazine equivalent dose*, ethnicity, smoking status	−3.13 (−6.84; 0.58), −0.005 (−0.01; −0.001)	0.098, 0.029
PANSS negative	gPM*. Chlorpromazine equivalent dose*	9.097 (1.98; 16.22), 0.007 (2e-4; 0.015)	0.014, 0.047	pPT, number of treatment drugs for psychosis, ethnicity*	−7.4 (−14.68; −0.12), 0.88 (0.16; 1.59)	0.053, 0.022
PANSS Positive	gNM*, gPM, number of medications total*, smoking status*	2.49 (0.191; 4.79), 5.36 (−0.57; 11.29), 0.63 (0.034; 1.22), 2.52 (0.095; 4.95)	0.036, 0.08, 0.041, 0.044	pPT, chlorpromazine equivalent dose, number of psychotic episodes		
SWN20	gPM, illness duration in years, number of medications total	−15.89 (−31.99; 0.2), −2.043 (−4.43; 0.34)	0.058, 0.099	pPT, age**, chlorpromazine equivalent dose*, illness duration in years*, number of psychotic episodes, smoking status	−2.385 (−3.47; −1.3), −0.036 (−0.063; −0.01), 2.072 (0.202; 3.94)	0.001, 0.017, 0.048
UPDRS	gPM*, sex*, chlorpromazine equivalent dose*, use of birth control	0.27 (0.056; 0.48), −0.14 (−0.27; −0.011), 3e−04 (6e-05; 0.001), 0.20 (0.003; 0.40)	0.015, 0.036, 0.018, 0.05	gPT, sex*, age**, chlorpromazine equivalent dose, illness duration in years, use of birth control	−0.28 (−0.53; −0.036), 0.024 (0.012; 0.036), 0.28 (0.003; 0.56)	0.03, 0.001, 0.055

AIMS: abnormal involuntary movement scale; BARS: Barnes Akathisia rating scale; PANSS: positive and negative syndrome scale; SWN20: subjective well-being under neuroleptics scale; UPDRS: unified Parkinson’s disease rating scale; pPT: phenoconversion-corrected phenotype; gPT: genotype-predicted phenotype; gPM: genotype-predicted poor metabolizer; gNM: genotype-predicted normal metabolizer.

**Table 7.** Predictors included in final models for *CYP2D6* non-victim drugs olanzapine and clozapine. Predictors with *p* < 0.01 are in italics and are marked with two asterisks, predictors with *p* < 0.05 have a single asterisk. Predictors with *p* < 0.1 are in italics only. Predictors with *p* > 0.1 are in standard lettering, without effect coefficients.

	Olanzapine	Effect size (95% CI)	*p*-Value	Clozapine	Effect size (95% CI)	*p*-Value
AIMS	pPT, age, chlorpromazine equivalent dose*	−0.003 (−0.005; 3e-04), 2e-04 (4e-05; 3e-04)	0.081, 0.012	pNM*, pPM, chlorpromazine equivalent dose, number of treatment drugs for psychosis total	−0.108 (−0.203; −0.013), −0.102 (−0.22; 0.012)	0.03, 0.085
BARS	gNM**, illness duration in years, ethnicity	1.66 (0.58; 2.73), 0.16 (−0.022; 0.33), −0.18 (−0.38; 0.017)	0.003, 0.087, 0.073	pPT, number of treatment drugs for psychosis, number of medications total*, ethnicity, smoking status	−0.44 (−0.83; −0.038), −0.26 (−0.52; −3e-05)	0.032, 0.05
PANSS negative	pPT, sex*, age, number of psychotic episodes*, smoking status	−4.254(−7.64; −0.87), −1.41 (−2.79; −0.04), −2.48 (−5.21; 0.25)	0.015, 0.047, 0.078	pPT		
PANSS Positive	pNM, sex, chlorpromazine equivalent dose	2.35 (−0.35; 5.047), −2.59 (−5.48; 0.3)	0.09, 0.081	pPT, sex**, age*, number of medications total, smoking status	8.64 (2.88; 14.4), 0.42 (0.094; 0.74), 3.703 (0.043; 7.36)	0.005, 0.015, 0.053
SWN20	pPT, sex, age*, number of treatment drugs for psychosis	9.96 (−0.014; 19.93), −0.55 (−1.038; −0.064), −18.65 (−37.31; 0.019)	0.054, 0.03, 0.054	pPM, chlorpromazine equivalent dose, smoking status*	−12.97 (−25.68; −0.27), 0.021 (0; 0.042), −10.46 (−19.53; −1.39)	0.055, 0.056, 0.031
UPDRS	gPT, sex**, smoking status, use of birth control*	0.26 (0.09; 0.44), −0.104 (−0.22; 0.009), −0.29 (−0.56; −0.015)	0.004, 0.074, 0.041	gPT, age, number of psychotic episodes*, number of treatment drugs for psychosis	0.088 (0.015; 0.16)	0.022

AIMS: abnormal involuntary movement scale; BARS: Barnes Akathisia rating scale; PANSS: positive and negative syndrome scale; SWN20: subjective well-being under neuroleptics scale; UPDRS: unified Parkinson’s disease rating scale; pPT: phenoconversion-corrected phenotype; gPT: genotype-predicted phenotype; gPM: genotype-predicted poor metabolizer; gNM: genotype-predicted normal metabolizer.

The selection of predictors in the final model for each outcome differed between drug groups. Higher chlorpromazine-equivalent dose, number of medications, and age, as well as being a smoker and of non-white ethnicity, were consistently associated with worse outcomes (see Tables 6 and 7). For illness duration, number of psychotic episodes, use of birth control and sex, and direction of effect differed between drug groups. Longer illness duration in years was associated with worse AIMS outcome in the risperidone group (*p* = 0.003), but improved outcome in the aripiprazole group (lower AIMS; *p* = 0.03, and higher SWN20; *p* = 0.048). A number of psychotic episodes, however, was associated with increased AIMS (aripiprazole; *p* = 0.03) and UPDRS scores (clozapine; *p* = 0.022), but lower PANSS negative scores (olanzapine; *p* = 0.047). The use of contraceptives was only associated with higher AIMS scores (aripiprazole; *p* = 0.031) but lower UPDRS scores (olanzapine; *p* = 0.041). Sex was associated with both PANSS scales yet in different groups (negative, olanzapine; *p* = 0.015, positive, clozapine; *p* = 0.005). It was also significant for the final models for UPDRS for risperidone (*p* = 0.036), olanzapine (*p* = 0.004), and aripiprazole (*p* = 0.03), though the model predicted increased UDPRS scores for women in the olanzapine group only, compared to lower scores in the others.

## Discussion

The aim of this paper was to investigate the frequency of phenoconversion and examine whether gPT or pPT was more often associated with side effects and PANSS scores. We found no significant differences between gPT groups or pPT groups in any of the outcome measures in the aggregated group, with exception of the pPT and the PANSS positive scale. This was likely driven by the risperidone group, as there were no between-phenotype group differences on any outcome for any other drug group. Across drugs, pPT was included in the final model for most, but not all, measures. Furthermore, only the AIMS and UPDRS were associated with pPT and gPT, respectively, in all subgroups. Phenotype was not significantly associated with the outcome for all drugs for any measure. The similarity in AIC for gPT and pPT models for some outcome measures also indicates that pPT is not superior to gPT to improve model fit in these cases.

*CYP2D6* substrate (‘victim’) drugs risperidone and aripiprazole were expected to be most affected by *CYP2D6* phenotype. Risperidone was indeed the only drug for which phenotype was associated with multiple measures (PANSS negative: gPM estimate = 9.097, *p* = 0.014; PANSS positive: gNM estimate = 2.49, *p* = 0.036; UDPRS: gPM estimate = 0.27, *p* = 0.015), and the only subgroup for which between-phenotype group differences were found (PANSS positive scale, *p* = 0.0286). However, aripiprazole was unaffected by *CYP2D6* genotype and phenoconversion. Although aripiprazole is included in the DPWG guidelines suggesting reduced dose for PMs, as phenotype appears to be correlated to plasma concentration of the sum of aripiprazole and dehydroaripiprazole, there is insufficient or no evidence for an effect on clinical effect of adverse reactions (Beunk et al., 2023). Similarly, study findings on *CYP2D6* phenotype and risperidone adverse reactions are mixed (de Brabander et al., 2024). Contrary to expectations, *CYP2D6* phenotype was significantly associated with one model for each olanzapine and clozapine. gNM individuals in the olanzapine group, and pNM individuals in the clozapine group, had better outcomes on the BARS and AIMS (respectively) compared to g/pIM and g/pPMs. The effect coefficients were small, however, and other factors such as sex and smoking status, contributed to a greater extent. This is within the realm of expectations, as they can affect *CYP1A2* activity, which plays a main role in the metabolism of both drugs (Carrillo et al., 2003; Fekete et al., 2023; Ou-Yang et al., 2000). Our finding may further confirm a limited effect of *CYP2D6* in adverse reactions. Alternatively, *CYP2D6* metabolizer status affects specific outcomes not appraised in this study. Previously, den Uil et al. (2023) demonstrated no difference between *CYP2D6* phenoconverted phenotype on side effects in general but did report an increase in specific side effects in PM and IM individuals. In both cases, this would also explain the lack of association between phenoconversion and outcome. If *CYP2D6* activity has no relationship with treatment outcome of drugs for psychosis in general, it would be expected that a change in activity through phenoconversion has no effect either.

Other aspects of the study itself may also explain our findings. Here, aripiprazole presented the smallest subgroup of *n* = 49, with 3 gPMs and 14 pPMs. Although this was the highest percentage of gPM and pPM in any group (6.2% and 28.6%, respectively), gPT and pPT proportions did not significantly differ between drug groups (Table S3). In general, the relatively small subsample of 51 patients for which phenoconversion occurred may have contributed to the lack of phenoconversion effect. Although our findings highlight the high frequency of phenoconversion and subsequential increase in pPMs, our sample may lack statistical power. The large increase in PM individuals following phenoconversion may have increased within-group variability, potentially distorting the results. With greater variability, statistical power decreases, and the sample size may not have been large enough to compensate for this (Norton and Strube, 2001). Furthermore, there may be patient-related factors associated with the use of concomitant medication that affected the outcome measures on their own. pPM individuals were either gPM or treated with SERTs paroxetine or fluoxetine. Reason for use of this medication, or their associated effect, may have interacted with treatment outcome (Bertelsen et al., 2003; Ensom et al., 2001; Liston et al., 2002; Korver et al., 2012; van Alphen et al., 2012; van Duin et al., 2005).

Alternatively, patients were taking medication for a longer time and were already prescribed a dose minimizing side effects. The average duration of illness was 4.3 (±3.5) years with 1.7 (±1.03) psychotic episodes, possibly indicating that patients started treatment before the first study measure was collected. Users of clozapine had a significantly longer illness duration of (6.2 ± 2.8) years, which may reflect its status as the first choice for treatment-resistant schizophrenia. In the Netherlands, clozapine is prescribed when treatment with two previous drugs for psychosis fails (van Alphen et al., 2012; van Duin et al., 2005). Although a subset of patients does not respond well to pharmacotherapy, the majority of patients with a first episode of psychosis respond sufficiently to treatment (Agid et al., 2013; Cheng et al., 2020; Gómez-Revuelta et al., 2020; Üçok and Kara, 2020; Wold et al., 2023). In addition, earlier studies found that olanzapine may be associated with later discontinuation and thus longer successful treatment (Agid et al., 2013; Gómez-Revuelta et al., 2020; Keating et al., 2021; Swartz et al., 2008). In this sample, 35.7% of patients were using olanzapine as their main treatment drug, and 17.5% used clozapine. Patient stability may thus have masked between-phenotype group outcomes.

Concerning the impact of phenoconversion, we found that approximately 25% of patients in the complete sample reported using a *CYP2D6* inhibitor, of which 68% used a strong inhibitor (paroxetine or fluoxetine). In all, 65 instances of phenoconversion from IM or NM to PM occurred, 51 of which were in the analysed sample, resulting in an increase in PM by almost five times (82 vs 17, 20% of the total sample). This high prevalence seems to be in line with previous literature and confirms that the PM phenotype could be more often caused by phenoconversion than by genetic predisposition (den Uil et al., 2023; Mostafa et al., 2019; Preskorn et al., 2013). In addition, it highlights the potential effects of polypharmacy. On average, participants reported use of about three medications (3.2 ± 2.04). Clozapine users reported the highest number of concomitant medications (4.3 ± 2.2). Although the number of medications was significant only for BARS (clozapine) and PANSS positive (risperidone) scores, when included in a model it was always associated with worse outcomes. Polypharmacy is common in psychiatry and may be associated with worse treatment outcomes, especially increased risk of side effects (Längle et al., 2012; Lähteenvuo and Tiihonen, 2021; Stassen et al., 2022a, 2022b). However, it can be useful in specific situations, depending on the presentation of symptoms or the severity of side effects. Concomitant prescription of drugs for depression such as SERTs is common to treat negative symptoms, although the reported effect is often small (Helfer et al., 2016; Galderisi et al., 2021; Lako et al., 2012; Mao and Zhang, 2015; Puranen et al., 2023; Singh et al., 2010; Terevnikov et al., 2015). ‘Antipsychotic polypharmacy’, when two or more drugs for psychosis are prescribed, may even be more effective than monotherapy without increased risk of side effects (Lähteenvuo and Tiihonen, 2021; Taipale et al., 2023; Tiihonen et al., 2019). In our results, the number of treatment drugs for psychosis was included in some final models, suggesting improved outcomes with antipsychotic polypharmacy. For no model was this significant, however.

In our sample, polypharmacy was slightly more prevalent in women. Although no significant differences were found between phenoconversion frequency, number of medications or inhibitor use (see Table S4), women appeared to be slightly more at risk of phenoconversion. 18.3% of women and 14.2% of men in the aggregated sample experienced this phenomenon. Women were prescribed more medications on average (3.6 vs 3.2) but were not more likely to report taking more than one medication. It has been suggested that there are sex-related differences in side effect outcomes, and women are at increased risk of overdosing (Hoekstra et al., 2021). Our results may suggest that women using olanzapine with lower *CYP2D6* activity (pPM/gIM compared to pNM/gNM) experienced fewer parkinsonian side effects but were at increased risk for akathisia. Such a between-phenotype result was not found for men. Sex itself was significant in the final model for UPDRS in the risperidone, olanzapine and aripiprazole groups. Women using aripiprazole or risperidone were predicted to have lower UPDRS scores, and the opposite if they were taking olanzapine. This may be due to the greater number of women non-smokers in this group, as smoking was (non-significantly) associated with reduced UDPRS scores. Differential effect of sex between drugs may also be related to sex-related differences in pharmacokinetics and pharmacodynamics, for example, levels of gastric acidity, body weight and distribution of adipose tissue, blood volume, degree of intestinal motility and renal excretion rates (Romanescu et al., 2022; Seeman, 2021). In addition, although no sex differences have been described for *CYP2D6*, evidence exists for increased activity of *CYP3A4* in women of reproductive age (Wolbold et al., 2003), and *CYP1A2* has been shown to vary with menstrual cycle (Jeong, 2010). The use of contraceptives was associated with lower UPDRS scores in the olanzapine group, which may be related to reduced *CYP1A2* fluctuations as olanzapine is primarily metabolized through this enzyme (Fekete et al., 2023). Evidence on the effect of hormonal contraceptives, especially non-oral, on psychotropic drugs is limited (Berry-Bibee et al., 2016), and it should be noted that the reported use of contraceptives in our sample was low. This is not uncommon in women with schizophrenia (Seeman and Ross, 2011). The association found here may suggest a potential interaction with the treatment effect.

Our findings may have several implications for clinical practice. The GROUP cohort is a well-characterized real-world sample of patients with a psychotic disorder, unconstrained by the inclusion criteria often specified for randomized clinical trials. This provides a more generalizable sample for clinical practice. It should be noted, however, that these patients do not represent severely ill and uncooperative patients with schizophrenia spectrum disorders. The study also demonstrates another important part of clinical reality that is often ignored in pharmacogenetic studies, the potential impact of polypharmacy and phenoconversion. This information is commonly ignored or included as a confounding variable only. Yet in our sample and practice, a large proportion of psychiatric patients are prescribed multiple medications (Kukreja et al., 2013). We have also demonstrated that polypharmacy leads to phenoconversion in a significant number of patients, which may affect treatment outcomes under certain conditions. The results also indicate, however, a limited role of *CYP2D6* activity in treatment outcome. Although *CYP2D6* genotyping is not yet standardized in clinical practice, we suggest that consideration of drug–drug interactions should be integral for patient treatment when pharmacogenetic information is available. Genotyping patients for relevant pharmacogenes should be supplemented by an indexation of concomitant medications possibly interfering with enzyme activity. Polypharmacy itself should be carefully considered, especially when new medications are added to the treatment regime. When a prescribing physician considers supplementing the treatment of drugs for psychosis with, for example, drugs for depression, they should review whether the new medication interferes with the treatment medication. Without such consideration, the patient may unnecessarily suffer from changes in the side effects or efficacy of the primary medication. If genotype information is available, phenoconversion should be integrated into standard treatment protocols through the use of regular updating of the predicted phenotype after changes in medication, depending on the inhibition or induction strength of other medications. A practical recommendation for this is the use of the University of Florida’s (UF Health) *CYP2D6* Phenoconversion (PROP Pharmacogenetics) Calculator developed by Cicali et al. (2021). This calculator is available online and is accessible also without knowledge of pharmacogenetics (https://precisionmedicine.ufhealth.org/how-to-interpret-results/phenoconversion-calculator/). This individual phenotype, rather than the genotype, should then be guiding medication choice and dose to optimize outcome.

Our study has some limitations that should be considered. First, the sample size of PMs was small, and it was not possible to analyse UMs due to the constraints of the GWAS panel. Since this metabolizer type is relatively rare, only a small number of UMs are expected to be missed (Kane, 2021). This should not have affected phenoconversion calculation, as UM individuals are also expected to convert to PM during the use of potent *CYP2D6* inhibitors, but does affect genotype-predicted estimations. Second, the number of women in the study was low. This is especially important to take into consideration when interpreting sex-related findings. The overrepresentation of men in schizophrenia and psychosis studies is not uncommon (Longenecker et al., 2010). The low number of women included in the study may be associated with the study design or recruitment methods; patients were recruited through clinicians. Previous research suggests that sex differences exist in the presentation of symptoms, as well as subsequential age of diagnosis and treatment (Barajas et al., 2015; Ferrara et al., 2024; Li et al., 2016). Possibly, fewer women were considered for potential inclusion due to bias in the recruitment methods. Alternatively, there may be other reasons for lowering the motivation of women to participate. As a result, the findings are not adequately powered to draw sex-specific conclusions about the impact of *CYP2D6* and phenoconversion, especially for women. Third, information on lifestyle factors, such as BMI, availability of a social support system, or pre-medication functioning, possibly contributing to treatment outcome, were unavailable at baseline. A lack of social support has also been associated with nonadherence (Rabinovitch et al., 2009). Nonadherence itself is a known challenge in psychotic disorders and has been associated with variability in treatment outcomes (Bozzatello et al., 2019; El Abdellati et al., 2020). Fourth, medication use was reported by the patient. Although all medication entries were manually checked and standardized prior to the current study, errors through patient reports or researcher documentation may have occurred, leading to incomplete records, inaccurate reports of dosage or omission of medication. This was considerably higher for documentation of the non-treatment medication. Concomitant inhibitor dose and duration of treatment could not be assessed, though these factors may affect phenoconversion. Although fluoxetine-induced *CYP2D6* inhibition has been shown to persist after cessation of fluoxetine administration, the effect is shorter for paroxetine (Bertelsen et al., 2003; Deodhar et al., 2021; Liston et al., 2002). The strength of inhibition may also increase with dose, although a low dose of 5 mg of paroxetine has been shown to inhibit *CYP2D6* activity already (Jeppesen et al., 1996; Jessurun et al., 2021). If a patient failed to report using either of these strong inhibitors at a very low dose or was not using the inhibitor at the time despite reporting to do so, they may have been wrongly considered pPM. A related limitation is the lack of therapeutic drug monitoring (TDM) in this sample. TDM is often used in clinical practice to optimize dose in accordance with the therapeutic windows of a drug and can be used to assess medication adherence and *CYP2D6* phenotype, as blood plasma concentration of the drug is expected to be higher for PM individuals (Ensom et al., 2001). However, this is not common practice for psychosis treatment in the Netherlands, where psychosis treatment guidelines specify no need for routine TDM due to a lack of evidence for reliable therapeutic ranges and it is employed only for clozapine and haloperidol when there are signs of treatment resistance or lack of response (van Alphen et al., 2012; van Duin et al., 2005). In the GROUP study from which the data were derived, medication compliance was assessed by a clinician using a 7-point Likert scale (Korver et al., 2012). Thus, there is a possibility that patients were erroneously categorized as compliant, leading to an incorrect classification as pPM (if nonadherence affected the inhibitory medication) or to the incorrect association of the treatment medication with outcome measures (if nonadherence affected treatment). Similarly, phenotype could only be predicted using genotype and was not confirmed using plasma drug concentration through TDM or using probe-specific xenobiotics and urine or blood sampling, as neither method was part of the study protocol nor standardized treatment in The Netherlands (Bozzatello et al., 2019; El Abdellati et al., 2020). However, genotype–phenotype concordance was previously found to be high for all but UM phenotype, suggesting this problem may have been minimized in the current sample (Ing Lorenzini et al., 2021). Regardless, without information on plasma concentration and complete medication regime of treatment and inhibitory drugs, we cannot assure that phenotype prediction, whether based on genotype or phenoconversion, was correct. Last, only *CYP2D6* phenotype and inhibitors were considered. Olanzapine and clozapine are mostly metabolized through flavin mono-oxygenase 3 (olanzapine), *CYP3A4* (clozapine) and *CYP1A2* (both) (Callaghan et al., 1999; Eiermann et al., 1997; Thorn et al., 2018), which may also be affected by concomitant medication. In addition, *CYP2D6* inhibitors are influenced by other enzymes. Paroxetine is a substrate of *CYP2D6* and *CYP3A4*, and fluoxetine is metabolized by several CYP enzymes including *CYP2C9, CYP2C19, CYP2D6* and *CYP3A4* (Jornil et al., 2010; Mandrioli et al., 2006). Non-NM status for these enzymes may affect their pharmacokinetic and -dynamic properties (Daly, 2017; English et al., 2012). For example, increased metabolization of *CYP2D6* inhibitors may affect their strength, and patients are classified erroneously as phenoconverted.

These limitations could be overcome in future studies utilizing larger samples, in diverse population. A suggestion to include more women may be to include older participants, as women tend to be diagnosed later than men (Ferrara et al., 2024; Sommer et al., 2020). Implementing a standardized TDM protocol into the study design would benefit the ascertainment of phenotype, and future studies should ensure thorough documentation of medication use, dose and duration of treatment. Prescription reporting through pharmacies could be used in addition to patient interviews to assess medication regimes. Furthermore, using modern approaches, data analysis allowing for the examination of copy number variants to categorize UM individuals would be of interest. Other *CYP* enzymes, such as *CYP1A2* and *CYP3A4* may be included in future analysis and their activity could be used to more accurately estimate the inhibitory activity of concomitant medication. Pharmacogenetic studies should always include polypharmacy and phenoconversion in their analysis and report pPT as well as gPT in their results.

We conclude that the prevalence of phenoconversion was high in the GROUP cohort and accounted for a significant increase in PM status due to concomitant use of SERTs. Neither *CYP2D6*-predicted nor phenoconversion-corrected phenotype was robustly associated with outcome measures. Risperidone, however, was affected most by *CYP2D6* genotype. The results suggest that PM phenotype is more often caused by phenoconversion than genotype, signifying the importance of phenoconversion in this psychiatric sample.

## Supplemental Material

sj-docx-1-jop-10.1177_02698811241278844 – Supplemental material for Clinical effects of CYP2D6 phenoconversion in patients with psychosis Supplemental material, sj-docx-1-jop-10.1177_02698811241278844 for Clinical effects of CYP2D6 phenoconversion in patients with psychosis by Emma Y De Brabander, Esmee Breddels, Therese van Amelsvoort and Roos van Westrhenen; GROUP Investigators in Journal of Psychopharmacology
