# Supplementary material for: Clinical effects of CYP2D6 phenoconversion in patients with psychosis
Source: J Psychopharmacol. 2024 Sep 23;38(12):1095–110. doi: 10.1177/02698811241278844 (PMC11528948; doi:10.1177/02698811241278844)
Supplement: sj-docx-1-jop-10.1177_02698811241278844 – Supplemental material for Clinical effects of CYP2D6 phenoconversion in patients with psychosis [file sj-docx-1-jop-10.1177_02698811241278844.docx]

**Supplemental material**

**Contents**

|  | Page |
| --- | --- |
| **Supplementary methods:** Details genotyping, quality control, and imputation. | **2** |
| **Supplementary table S1:** CYP2D6 inhibitors. | **3** |
| **Supplementary table S2**. Demographic variables complete sample. | **4** |
| **Supplementary table S3**. Between drug-group differences. | **6** |
| **Supplementary table S4.** Between sex differences (of aggregated group, n = 342). | **7** |
| **Supplementary table S5.** Sample size per drug group per outcome. | **7** |
| **Supplementary table S6**. AIC and R^2^ for each final model. | **8** |
| **Supplementary Figure S1**. Conversion from genotype-predicted phenotype (gPT) to phenoconversion-corrected phenotype (pPT) depending on inhibitor strength according to Cicali et al. (2021). | **9** |

**Supplementary methods: Details genotyping, quality control, and imputation.**

This protocol has been adapted from Sandhu et al. (2023).
Genotype data was available for 2812 individuals (including patients, siblings, parents, and healthy controls) and 570038 SNVs. This data was generated on a customized Illumina Institute of Psychological Medicine and Clinical Neurology assay. The chips contained ~250K common variants and 250K exome chip variants (rate, exomic, nonsynonymous, MAF <1%), as well as ~50K variants relevant for psychiatric indications.

SNVs and samples with call rates below 95% (SNV) and 98% (sample) were removed. Following this, SNVs with a MAF threshold of <10% and Hardy-Weinberg Equilibrium *p*-value <1e-0.5 were removed. SNV pruning based on linkage disequilibrium (LD) of R2 <0.2, with window size 50 and window shift per 5 SNVs was performed next.

~58K SNVs remained to assess sex errors, heterozygosity (F <3 SD), homozygosity (F > 3 SD), and relatedness (pairwise identity by descent values). Values that were considered duplicated (identical pihat >0.8) were also removed, and the remaining pairs were manually checked since the sample included family members. Any failed samples were also removed.

Next, SNVs with a call rate below 98% were removed again.

Ancestry was investigated using multidimensional scaling clustering with Hapmap phase 3. The first 10 principal components were calculated using EIGENSTRAT software (<https://github.com/DReichLab/EIG>) and samples deviating more than 3SD from the White/Caucasian ancestry sample in the first 4 components were removed.

Limited quality control was then performed again: SNV call rate > 95% and sample call rate >98%. SNVs with MAF <1 remained, to assess rare variants.

2503 individuals and 560K SNVs remained.

Pre-imputation checks were performed based on the imputation reference panel, before preparation and imputation on the Michigan Imputation Server (https:// (https://imputationserver.sph.umich.edu) using Minimac

4. 1000 Genome Phase 3 (version 5) reference panel (build hg19, population: Mixed). To include only high quality rare variants, a post imputation filter of R2 > 0.5 was employed.

| **Weak** | **Moderate** | **Strong** | **In-vitro evidence only** | **Strength level under review** |
| --- | --- | --- | --- | --- |
| Amiodarone | Abiraterone | Bupropion | Chlorpromazine | Methadone |
| Celecoxib | Cinacalcet | Fluoxetine | Clemastine | Midodrine |
| Cimetidine | Clobazam | Paroxetine | Cocaine | Panobinostat |
| Citalopram | Doxepin | Quinidine | Haloperidol | Promethazine |
| Clomipramine | Duloxetine |  | Hydroxyzine |  |
| Diphenhydramine | Halofantrine |  | Metoclopramide |  |
| Escitalopram | Lorcaserin |  | Perphenazine |  |
| Hydroxychloroquine | Moclobemide |  | Ticlopidine |  |
| Levomepromazine | Rolapitant |  | Tripelennamine |  |
| Ritonavir |  |  |  |  |
| Sertraline | Terbinafine (oral) |  |  |  |
| Vermurafenib |  |  |  |  |

Table S1. CYP2D6 inhibitors. Adapted from Flockhart et al. (2021); accessed 13-11-23.

|  | | **Total** | **Men** | **Women** |
| --- | --- | --- | --- | --- |
| **Patients (% of total)** | | 412 (100%) | 328 (79.6%) | 82 (20.4%) |
| **Average age in years*** | | 27.2 (± 7.1) | 26.8 (± 6.2) | 29.1 (± 9.7) |
| **Average number of total medications** | | 3.28 (± 2.03) | 3.2 (± 1.9) | 3.7 (± 2.1) |
| **Average duration of illness in years** | | 4.36 (± 3.4) | 4.6 (± 3.5) | 4.01 (± 3.3) |
| **Number of psychotic episodes** | | 1.8 (± 1.1) | 1.7 (± 1.1) | 1.8 (± 1) |
| **Ethnicity** | White | 338 (82.8%) | 268 (82.5%) | 70 (84.3%) |
|  | Moroccan | 13 (3.2%) | 12 (4%) | 0 |
|  | Turkish | 12 (2.9%) | 11 (3.4%) | 1 (1.2%) |
|  | Other | 10 (2.5%) | 7 (2.2%) | 3 (3.6%) |
|  | Mixed | 35 (8.6%) | 26 (8%) | 9 (10.8%) |
| **Diagnosis** | Schizophrenia and related disorders (including schizophreniform and schizoaffective) | 357 (86.9%) | 288 (87.8%) | 69 (82.1%) |
|  | Bipolar disorder | 2 (0.5%) | 1 (0.3%) | 1 (1.2%) |
|  | Delusional disorder | 10 (2.4%) | 8 (2.4%) | 2 (2.4%) |
|  | Psychosis | 41 (10%) | 29 (8.8%) | 12 (14.3%) |
|  | Unknown | 1 (0.2%) | 1 (0.3%) | 0 |
| **Smoking status **** | Smoker | 258 (62.6%) | 221 (67.4%) | 37 (44%) |
|  | Non-smoker | 147 (35.7%) | 101 (30.8%) | 46 (54.8%) |
|  | Unknown | 7 (1.7%) | 6 (1.8%) | 1 (1.2%) |
| **Reported use of birth control (oral birth control and IUD)** | | 24 (5.8%) | NA | 24 (28.6%) |
| **Main treatment drug** | Olanzapine | 122 (29.7%) | 101 (30.8%) | 21 (25%) |
|  | Risperidone | 111 (27%) | 91 (27.7%) | 20 (23.8%) |
|  | Clozapine | 60 (14.6%) | 53 (16.2%) | 7 (8.3%) |
|  | Aripiprazole | 49 (11.9%) | 37 (11.3%) | 12 (14.3%) |
|  | Quetiapine | 27 (6.6%) | 16 (4.9%) | 11 (13.1%) |
|  | Haloperidol | 15 (3.6%) | 12 (3.7%) | 4 (4.8%) |
|  | Flupentixol | 11 (2.7%) | 8 (2.4%) | 3 (3.6%) |
|  | Amisulpride | 8 (1.9%) | 5 (1.5%) | 3 (3.6%) |
|  | Zuclopentixol | 3 (0.7%) | 3 (0.9%) | 0 |
|  | Penfluridol | 3 (0.7%) | 2 (0.6%) | 1 (1.2%) |
|  | Perfenazine | 1 (0.2%) | 0 | 1 (1.2%) |
|  | Sulpiride | 1 (0.2%) | 0 | 1 (1.2%) |
| **Chlorpromazine-equivalent dose of main treatment drug (in mg) **** | | 358.7 (± 193) | 305.6 (± 171.3) | 372.4 (± 197.2) |
| **CYP2D6 inhibitors (strength)** | Paroxetine (Strong) | 60 (47.2%) | 45 (47.9%) | 14 (45.5%) |
|  | Citalopram (Weak) | 28 (22%) | 24 (25.5%) | 4 (12.1%) |
|  | Fluoxetine (Strong) | 14 (11%) | 10 (10.6%) | 4 (12.1%) |
|  | Sertraline (Weak) | 13 (10.2%) | 9 (9.6%) | 4 (12.1%) |
|  | Escitalopram (Weak) | 7 (5.5%) | 5 (5.3%) | 2 (6.1%) |
|  | Clomipramine (Weak) | 4 (3.1%) | 1 (1.1%) | 3 (9.1%) |
|  | Levomepromazine (Weak) | 1 (0.8%) | 0 | 1 (3%) |
| **CYP2D6 genotype predicted phenotype** | PM | 17 (4.1%) | 12 (3.7%) | 5 (6%) |
|  | IM | 167 (40.5%) | 126 (38.4%) | 41 (48.8%) |
|  | NM | 228 (55.3%) | 190 (57.9%) | 38 (45.2%) |
| **CYP2D6 phenoconversion corrected phenotype** | PM | 82 (19.9%) | 60 (18.3%) | 22 (26.2%) |
|  | IM | 129 (31.3%) | 98 (38.4%) | 31 (36.9%) |
|  | NM | 201 (48.8%) | 170 (51.8%) | 31 (36.9%) |

Table S2. Demographic variables complete sample. Between-sex differences are marked with two asterisks at p < 0.01 and with a single asterisk at p < 0.05. PM: poor metabolizer. IM: intermediate metabolizer. NM: normal metabolizer

| Variable | P-value | P-value follow-up tests |  |
| --- | --- | --- | --- |
| CYP2D6 genotype predicted phenotype | 0.28 |  |  |
| CYP2D6 phenoconversion corrected phenotype | 0.12 |  |  |
| Sex | 0.37 |  |  |
| Age | <0.001 | Risperidone vs olanzapine  Risperidone vs clozapine  Risperidone vs aripiprazole | <0.001  0.016  0.019 |
| Ethnicity | 0.56 |  |  |
| Smoking status | 0.29 |  |  |
| Reported use of birth control (oral birth control and IUD) | 0.009 | Clozapine vs aripiprazole | 0.007 |
| Total number of medications | <0.001 | Risperidone vs clozapine  Risperidone vs aripiprazole  Olanzapine vs clozapine  Olanzapine vs aripiprazole | <0.001  0.019  <0.001  0.019 |
| Total number of treatment drugs for psychosis | 0.002 | Risperidone vs olanzapine  Risperidone vs clozapine | 0.002  0.048 |
| Duration of illness in years | <0.001 | Risperidone vs clozapine  Risperidone vs aripiprazole  Olanzapine vs clozapine | <0.001  <0.001  <0.001 |
| Number of psychotic episodes | 0.14 |  |  |
| Chlorpromazine-equivalent dose of main treatment drug (in mg) | <0.001 | Risperidone vs olanzapine  Risperidone vs clozapine  Olanzapine vs aripiprazole | <0.001  <0.011  0.012 |
| CYP2D6 inhibitor use | 0.057 |  |  |

Table S3. Between drug-group differences. Continuous variables (age, total number of medications, total number of treatment drugs for psychosis, duration of illness in years, number of psychotic episodes, and chlorpromazine equivalent dose of main treatment drug) were examined using Kruskal-Wallis test and post-hoc Dunn test. Categorical variables (CYP2D6 genotype predicted phenotype, CYP2D6 phenoconversion-corrected phenotype, sex, ethnicity, smoking status, reported use of birth control, and CYP2D6 inhibitor use) were examined using Fisher’s Exact test and post-hoc pairwise Fisher’s test.

| Variable | P-value |
| --- | --- |
| CYP2D6 genotype predicted phenotype | 0.4 |
| CYP2D6 phenoconversion corrected phenotype | 0.17 |
| Age | 0.17 |
| Ethnicity | 0.33 |
| Smoking status | <0.001 |
| Total number of medications | 0.12 |
| Total number of treatment drugs for psychosis | 0.62 |
| Duration of illness in years | 0.38 |
| Number of psychotic episodes | 0.53 |
| Chlorpromazine-equivalent dose of main treatment drug (in mg) | 0.001 |
| CYP2D6 inhibitor use | 0.41 |

Table S4. Between sex differences (of aggregated group, n = 342). Continuous variables (age, total number of medications, total number of treatment drugs for psychosis, duration of illness in years, number of psychotic episodes, and chlorpromazine-equivalent dose of main treatment drug) were examined using Mann-Whitney U test. Categorical variables (CYP2D6 genotype predicted phenotype, CYP2D6 phenoconversion-corrected phenotype, ethnicity, smoking status, and CYP2D6 inhibitor use) were examined using Chi-Square test.

|  | AIMS | BARS | SWN20 | UPDRS | PANSS Positive | PANSS Negative |
| --- | --- | --- | --- | --- | --- | --- |
| Risperidone | 108 | 108 | 57 | 108 | 104 | 102 |
| Olanzapine | 116 | 117 | 79 | 117 | 112 | 113 |
| Clozapine | 58 | 57 | 35 | 57 | 555 | 56 |
| Aripiprazole | 45 | 46 | 22 | 46 | 45 | 46 |

Table S5. Sample size per drug group per outcome.

|  |  | AIMS | BARS | SWN20 | UPDRS | PANSS Positive | PANSS Negative |
| --- | --- | --- | --- | --- | --- | --- | --- |
| Risperidone | AIC | 38.03 | 191.81 | 460.89 | -4.35 | 662.34 | 663.051 |
|  | R^2^ | 0.13 | 0.033 | 0.18 | 0.17 | 0.16 | 0.107 |
| Olanzapine | AIC | -152.01 | 138.32 | 662.76 | 51.08 | 719.57 | 756.47 |
|  | R^2^ | 0.104 | 0.12 | 0.12 | 0.13 | 0.085 | 0.12 |
| Clozapine | AIC | -44.77 | 92.59 | 282.69 | 27.07 | 369.58 | 381.6 |
|  | R^2^ | 0.14 | 0.19 | 0.33 | 0.17 | 0.28 | 0.013 |
| Aripiprazole | AIC | -92.12 | 59.82 | 172.19 | 1.73 | 313.42 | 312.04 |
|  | R^2^ | 0.401 | 0.30 | 0.67 | 0.34 | 0.103 | 0.19 |

Table S6. AIC and R^2^ for each final model.


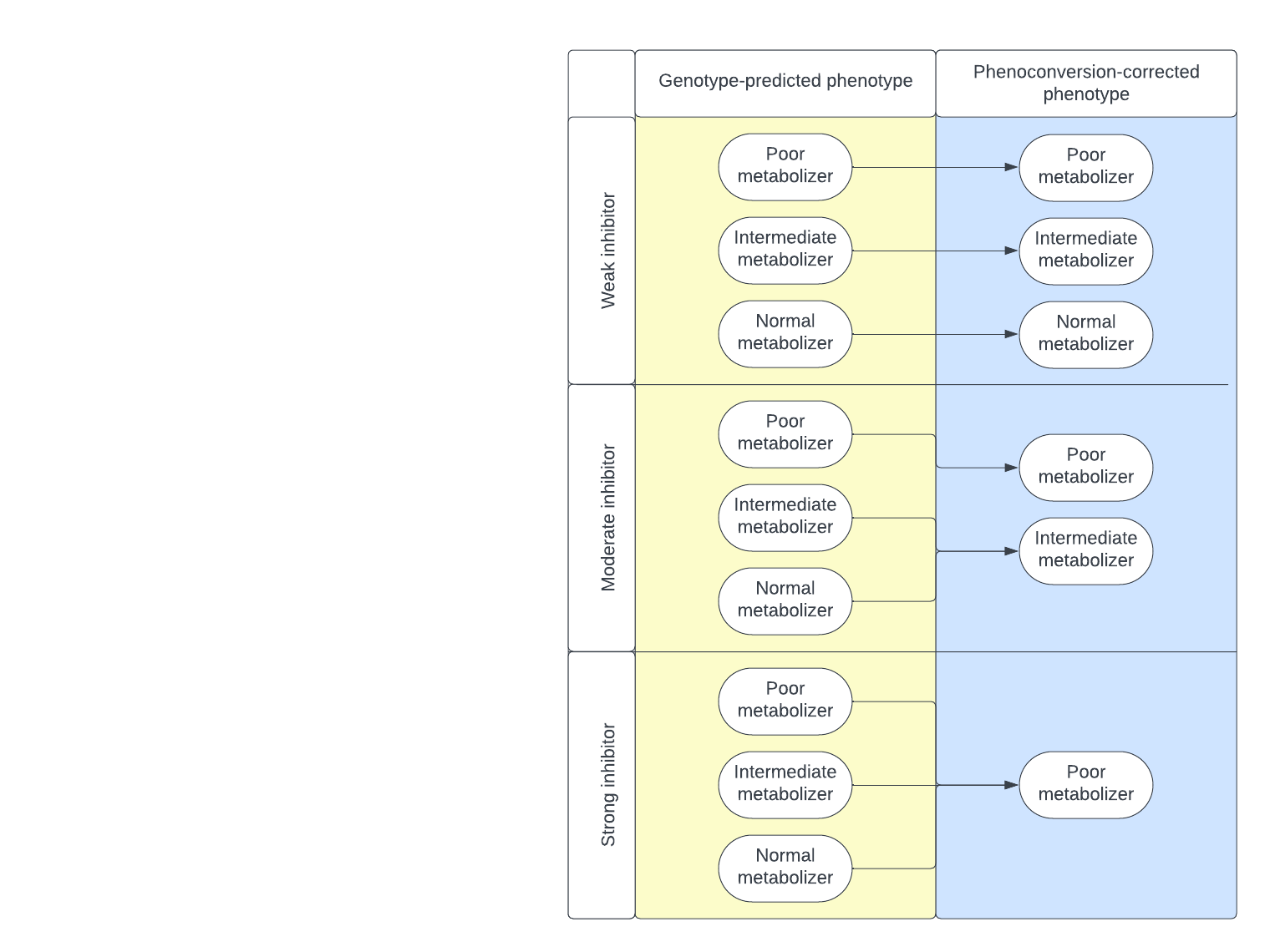


Figure S1. Conversion from genotype-predicted phenotype (gPT) to phenoconversion-corrected phenotype (pPT) depending on inhibitor strength according to Cicali et al. (2021).
